# Nurses' Workplace Social Capital and Sustainable Development: An Integrative Review of Empirical Studies

**DOI:** 10.1155/2024/8362035

**Published:** 2024-07-17

**Authors:** Jia-Min Xu, Ming-Guo Cao, Qian-Cheng Gao, Yi-Xuan Lu, Azadeh T. Stark

**Affiliations:** ^1^ Department of Nursing Sciences School of Medicine Lishui University, Lishui, China; ^2^ Department of Dentistry School of Medicine Lishui University, Lishui, China; ^3^ School of Interdisciplinary Studies University of Texas at Dallas, Richardson, TX, USA; ^4^ Department of Pathology and Laboratory Medicine Henry Ford Health, Detroit, MI, USA

## Abstract

**Aim:**

The purpose of our review was to assess the role of nurses' workplace social capital in meeting the Sustainable Development Goals (SDGs) of the United Nations (UN).

**Background:**

In 2015, the 2030 Agenda for Sustainable Development with 17 universal goals was adopted by members of the UN. Although nurses have been acknowledged as important contributors to sustainable development, they still have difficulties in connecting their work to the SDGs. Nurses' workplace social capital is an important concept in nursing management due to its constructive consequences. However, the potential association between nurses' workplace social capital and the SDGs has not been evaluated. *Evaluation*. We conducted an integrative review, following the methodology of Whittemore and Knafl. Seven databases, Medline, CINAHL, Web of Science, Cochrane Library, Embase, PsycINFO, and Scopus with no restriction on publication year, were searched in May 2023 to identify statistically significant empirical evidence. Only peer-reviewed research papers published in English language journals were considered. We applied the Mixed Methods Appraisal Tool to evaluate the quality of the selected articles. We categorized outcomes of nurses' workplace social capital into themes and connected them to the SDGs through repeated comparisons and discussions. *Key Issues*. Twenty-nine of 2,188 retrieved articles were included in the final data analysis. Twenty-three outcomes of nurses' workplace social capital were identified, and three themes were abstracted. Nurses' workplace social capital is positively associated with SDG 3 (good health and well-being), SDG 8 (decent work and economic growth), and SDG 17 (partnerships for the goals).

**Conclusion:**

Findings of our integrative review shed light on the importance of nurses' workplace social capital and the role of nurses in achieving the global movement for sustainable development. *Implication for Nursing Management.* Investment in nursing workforce and nurses' workplace social capital can further strengthen the position of nurses to support and deliver the SDGs.

## 1. Introduction

The concept of sustainability was introduced to the field of management in the 1950s because of the negative environmental impacts of human actions [1]. In September 2015, the 2030 Agenda for Sustainable Development by the UN endorsed 17 universal and transformative SDGs [2]. These 17 goals address economic, social, and environmental aspects of sustainable development. People, Planet, Peace, Prosperity, and Partnership (5Ps) form the pillars of the SDGs, with a shared vision to build a peaceful and healthy world [2, 3]. In 2020, the UN launched the Decade of Action plan, calling for the direct involvement of people, individually and collectively, to ascertain meeting the SDGs by 2030 [4].

Nurses carry a pivotal role in the sustainability movement of the healthcare industry. The statement by McMillan “*the profession of nursing claims no geographical boundaries, working in diverse areas of health care. These range from outpost and global nursing to areas in policy development. Such diverse job profiles position nursing at the heart of the sustainability movement in health care*” [[5], p.757] underlines the importance of nurses' roles and responsibilities in achieving sustainability. Additionally, the International Council of Nurses (ICN) has cited practical case examples from different countries to endorse the importance of nurses' contributions to the 17 SDGs [6]. Nurses, on a daily basis, contribute to some, if not most, of the 17 SDGs even though they might not be cognizant of their contributions. Their routine professional responsibilities are part and parcel of the activities that can achieve the goals of sustainable development [6, 7].

The concept of social capital can be traced back to the early 20^th^ century [8]. Workplace social capital, a concept under the umbrella term of social capital, explains the interactive relational networks in a workplace; it is the tenet of a healthy work environment that can protect employees' mental and psychological well-being and ensure their efficiency and effectiveness in delivering their professional responsibilities [9–11]. Workplace social capital has become more prominent in the healthcare industry because of the complexities of the delivery of healthcare services and therefore the interdependencies of healthcare professionals [12–14].

The concept of nurses' workplace social capital has been defined as “*a relational network configured by respectful interactions among nursing professionals and between the other healthcare professionals. These interactions are characterized by the norms of trust, reciprocity, shared understanding, and social cohesion*” [[14], p. 252]. Nurses' workplace social capital is a useful professional resource because it is a segue to a spectrum of positive outcomes for the healthcare industry, people, and the environment [13–15]. For example, nurses' workplace social capital has been attributed to safety and quality in the delivery of healthcare services, e.g., reduction of unnecessary duplication of services and/or medication errors or other interventions [16].

Does nurses' workplace social capital contribute to the SDGs of the UN? Despite the implicit evidence in practical cases [6], no research has been conducted to evaluate the association between these two pivotal concepts. Whittemore and Knafl [17] suggest that the approach of an integrative review allows bridging and connecting knowledge in providing a comprehensive and collective picture of a specific scientific phenomenon. In this integrative review, we attempt to demonstrate the association between nurses' workplace social capital and different domains of the SDGs based on empirical evidence. The results of our work should set the foundation for understanding the connection between nurses' workplace social capital and the SDGs; more importantly, our findings should shed further light on the critical roles of nurses in meeting the 2020 Decade of Action plan by the UN through their daily work responsibilities.

## 2. Methods

### 2.1. Study Design

We applied the integrative review approach of Whittemore and Knafl [17] to conduct our study. Five stages such as *Problem Identification, Literature Search, Data Evaluation, Data Analysis, and Presentation of the Findings* were implemented [17, 18].

### 2.2. Problem Identification

We reviewed the literature on social capital and sustainability to identify gaps in addressing the association between nurses' workplace social capital and the SDGs.

### 2.3. Literature Search Stage

Seven databases, Medline, CINAHL, Web of Science, Cochrane Library, Embase, PsycINFO, and Scopus, with no restriction on publication year, were searched to identify potential resources. In the initial stage of our search, we used the terms “social capital,” “sustainable development,” “sustainability,” “nurses,” and “nursing” with proper combination by the Boolean operators. Due to the limited number of identified publications, it became necessary to broaden the scope of our search; therefore, we used the terms “social capital,” “nurses,” “nurse,” and “nursing” to identify the potential resources. The details of our search can be found in Supplementary Table 1. The final systematic search was completed on May 31^st^, 2023. Additionally, we reviewed references of the selected papers to identify additional resources.

#### 2.3.1. Inclusion and Exclusion Criteria

We set three inclusion criteria: (1) empirical research papers that had assessed the direct or indirect association between nurses' workplace social capital and the SDGs; (2) English language publications; and (3) published in peer-reviewed journals. We did not discriminate on the method of empirical research, qualitative, quantitative, and mixed methods. We excluded empirical studies addressing patients' social capital, nursing students' social capital, and social capital among the other professionals. Furthermore, we eliminated studies that addressed social capital in circumstances other than the workplace; finally, we excluded studies of nurses' workplace social capital in which outcomes were not explicitly discussed.

#### 2.3.2. Search Outcomes

**Figure 1:**
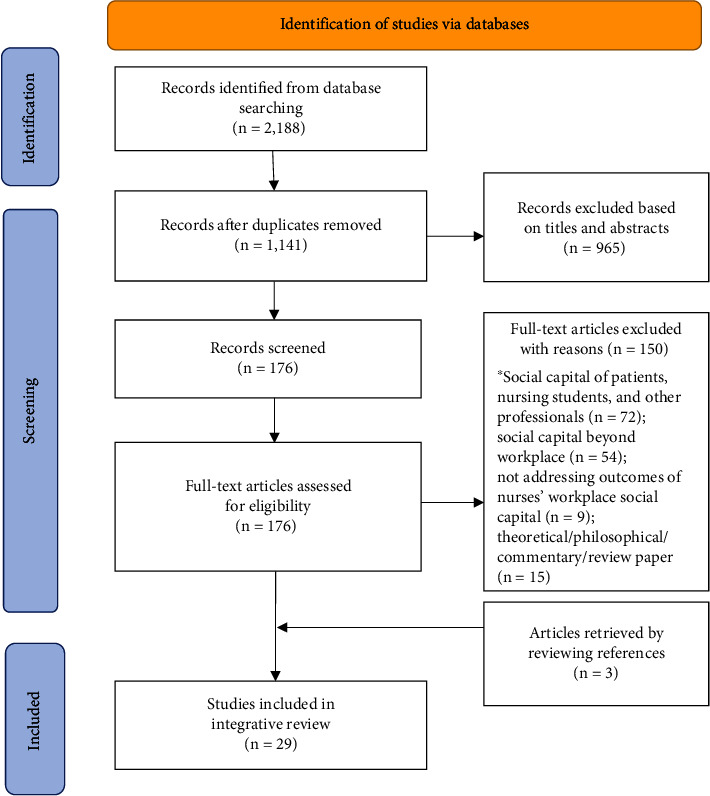
PRISMA flowchart for article selection.

An adapted PRISMA flow diagram was used to present the process and outcome of article selection [19] (Figure 1). Initially, a total of 2,188 potentially eligible records were identified from the selected seven databases. After removing duplicates using the EndNote software package, a total of 1,141 articles remained. The titles and abstracts of the remaining articles were then screened and a total of 176 articles remained for the full-text review. Of these, 150 articles were excluded for different reasons (Figure 1). Additionally, we reviewed references of the selected articles and retrieved three full-text papers. A total of 29 articles were included for the next stage, data evaluation. Two of the authors (QCG and YXL) conducted the literature search, while the screening process for meeting the inclusion criteria was performed by a third author (JMX). The result was confirmed by the team.

### 2.4. Data Evaluation Stage

We used the Mixed Methods Appraisal Tool (MMAT) to evaluate quality of the selected studies [20]. We justified the application of this tool because it is a comprehensive and a critical appraisal tool that can be used for different types of research including qualitative, quantitative, and mixed-methods studies (including instrument development and test) [20]. Initially, two of the authors (JMX and MGC) worked independently to critically evaluate the quality of the selected papers; disagreements were addressed by team discussions and by achieving a unanimous agreement among the authors. The details of the data appraisal are presented in Supplementary Table 2. No study was excluded in this process because the main purpose of our review was to integrate all empirical evidence of the contributions of nurses' workplace social capital to sustainable development. All the 29 identified articles were included in the next stage, data analysis.

### 2.5. Data Analysis Stage

**Figure 2:**
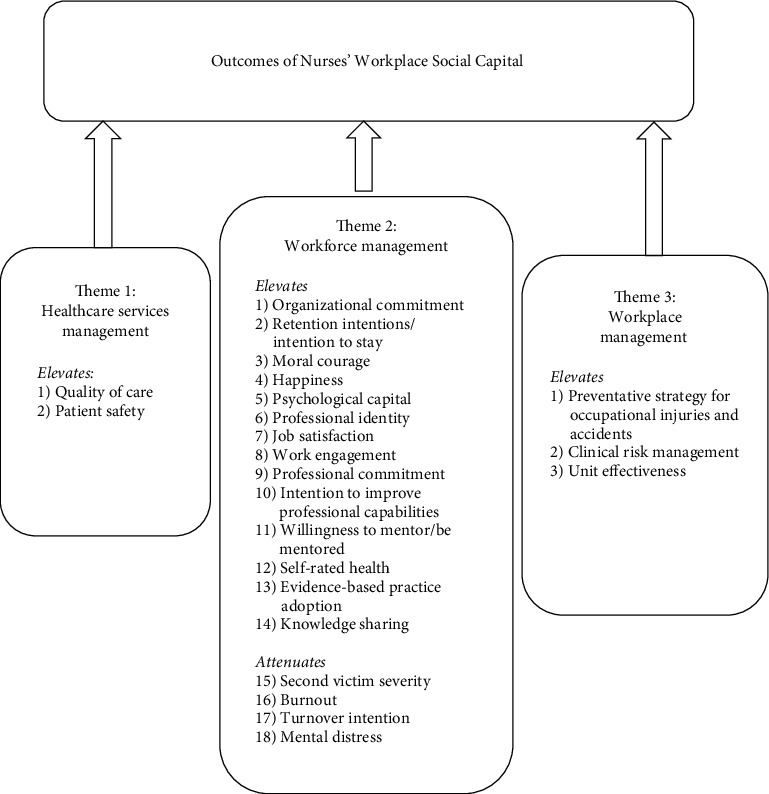
Emergence of three themes: healthcare services management, workforce management, and workplace management. Twenty-three statistically significant outcomes of nurses' workplace social capital reported by the 29 studies were grouped into three themes according to their characteristics and contributions.

The goal of data analysis in an integrative review is to summarize the data systematically and unbiasedly. The objective is to develop an innovative approach to synthesize data for the next stage which is the comprehensive presentation. The method of data analysis that we applied included data reduction, data display, data comparison, and drawing the conclusion and verification [17]. We developed a template to extract and display the characteristics of the selected studies; the variables that we included in categorizing the data were reference, study design, sample size, main findings, and instruments (Table 1). First, we coded the data and then grouped them under the appropriate variables. Under the variable of “main findings,” we classified the statistically significant outcomes of nurses' workplace social capital into themes (Figure 2). For example, the two reported outcomes “quality of care” and “patient safety” were clustered under the theme of “healthcare services management.” Finally, we applied the technique of repeated comparisons and discussions to link outcomes under each theme to the 17 SDGs (Table 2). Two of the authors (JMX and MGC) prepared the initial data extraction and data categorization which were then confirmed and improved by all team members.

## 3. Results

### 3.1. Study Characteristics

The characteristics of the 29 selected studies are presented chronologically by the year of publication (Table 1). A broad spectrum of sample size, ranging from 8 to 1,201, was identified among these studies. Ernstmann and colleagues were the first to empirically assess the association between nurses' workplace social capital and clinical risk management in hospitals [48]. Subsequently, a total of 28 other studies were implemented in different settings to assess the empirical association between nurses' workplace social capital and various positive outcomes in healthcare settings [16, 21–47].

Of the 29 selected studies, 24 were quantitative, two were qualitative, and the designs of the remaining three were mixed methods. The majority of the quantitative studies (*n* = 23) had a cross-sectional survey design. Only one study was designed as a longitudinal survey in which the investigators followed up newly employed nurses for one year to assess the impact of social capital on their job satisfaction and their mental health [40].

One of the two qualitative studies aimed to assess the value of nurses' workplace social capital in preventing occupational injuries and accidents in hospital settings [28]; while the second study was conducted among nurses who participated in management initiative training by the English National Health Service Trust [22]. Findings from the study asserted the positive influence of social capital networks and networking on nurses' retention intentions [22]. Finally, the result of our literature search yielded three mixed-methods studies. One was conducted among nurses in two university hospitals; the investigators explored the associations among several work-related variables and reported that emotional exhaustion, one dimension of burnout, was considerably reduced because of the workplace social capital; meanwhile, vigor, one dimension of work engagement, was increased [37]. The other two studies were implemented with the objective of developing and testing instruments to assess nurses' workplace social capital in different work environments with different workplace cultural norms and values [29, 43].

### 3.2. Outcomes of Nurses' Workplace Social Capital

We identified a total of 23 outcomes of nurses' workplace social capital. These outcomes were compared and inductively synthesized into three themes (Figure 2): healthcare services management (Theme 1), workforce management (Theme 2), and workplace management (Theme 3). Of the 23 identified outcomes, 18 were grouped under the theme of workforce management. We grouped quality of care and patient safety under the theme of healthcare services management. Finally, the theme of workplace management was summarized based on the three following outcomes: a preventative strategy for occupational injuries and accidents, clinical risk management, and unit effectiveness. It is notable that all the 23 outcomes were positive as reported in the selected empirical studies.

### 3.3. Nurses' Workplace Social Capital and the SDGs

Of all the 29 studies that were reviewed by the authors, none had directly assessed and described the association between nurses' workplace social capital and the SDGs. However, the implicit influence of nurses' workplace social capital on the SDGs was reported and discussed in these 29 studies. The implicit influence of nurses' workplace social capital was mostly reverberated on SDGs 3, 8, and 17 based on connections between its outcomes and SDGs (Table 2).

#### 3.3.1. Influence on Good Health and Well-Being (SDG 3)

Nurses are the critical contributors to attainment of SDG 3 [49, 50]. Quality of care and patient safety, which are the outcomes of nurses' workplace social capital (Theme 1) [16, 26, 38, 41, 45], are tightly linked to patients' good health and well-being. For instance, Van Bogaert et al. [16] reported that nurses' workplace social capital improves patients' safety through reduction in medication errors and improves the overall quality of care delivered to patients.

The five outcomes that are clustered under workforce management (Theme 2) demonstrate the influence of workplace social capital on health status of nurses, who are the backbone of provision of healthcare services. According to the findings by Middleton et al. [34], nurses with perception of low level of workplace social capital are at a higher risk for mental distress and compromised health status. The direct protective effect of workplace social capital on nurses' mental health was also reported by Read and Laschinger [40].

Burnout is a complex syndrome with multiple signs and symptoms. Improvement of nurses' workplace social capital is associated with feelings of increased personal accomplishment and decreased emotional exhaustion and depersonalization [26, 37, 39, 44, 47]. Diminished personal accomplishment, emotional exhaustion, and depersonalization have been cited as the core elements of burnout and the main cause of psychological and physical duress among nurses [26, 37, 39, 44, 47].

Self-perception of happiness is an important element for an individual's health and well-being. Pirdelkhosh et al. [23] reported that workplace social capital can be a positive facilitator of nurses' happiness in hospital settings during the stressful period such as COVID-19. The importance of strengthening nurses' workplace social capital in mitigating the possibilities of experiencing the second victimization, in case of an unanticipated adverse patient event, also has been documented [24].

Finally, the three outcomes of nurses' workplace social capital, a preventative strategy for occupational injuries and accidents at work, clinical risk management, and unit effectiveness, are classified under the theme of workplace management (Theme 3). These three outcomes are also in a close association with SDG 3 (good health and well-being). Social capital, in the form of social support, is instrumental in prevention of occupational injuries and accidents among nurses [28]. Nurses who possess a higher workplace social capital, in general, are more effective and perform more efficiently in managing clinical risk behaviors [33, 48]. Furthermore, unit work effectiveness in hospitals as perceived by nurses is also tightly linked to the positive effects of workplace social capital [41]. These outcomes of nurses' workplace social capital contribute to developing, implementing, and sustaining policies and procedures for a safe and constructive healthcare environment for patients and professionals. A constructive healthcare environment improves the overall health and permits good health and well-being for all (SDG 3).

#### 3.3.2. Influence on Decent Work and Economic Growth (SDG 8)

A total of 11 outcomes of nurses' workplace social capital under the theme of workforce management (Theme 2) are closely related to SDG 8 (Table 2). Investments in healthcare employment and the healthcare industry can improve economic productivity and economic growth [51]. Nurses are the backbone of delivery of healthcare services and the largest workforce in the healthcare industry. Unfortunately, in recent years the nursing profession has been grappling with the exodus of nurses from the field [52]. We identified a range of outcomes of nurses' workplace social capital (Theme 2) as the contributing factors in reducing, if not preventing, exodus and/or high turnover of nurses. Sheingold and Sheingold [43] and Van Bogaert et al. [16] reported that workplace social capital could increase nurses' intention to stay (no intention to leave). Fisher et al. [22] reported that social capital networks and networking at the workplace are positive factors that can influence nurses' retention intentions. Also, Norikoshi et al. [29] reported the protective effect of nurses' workplace social capital in ameliorating turnover intention. Job satisfaction is an important variable that can influence turnover and possibly could prevent the exodus of nurses from the field. Nurses' workplace social capital has been reported as a positive influencer of job satisfaction in several studies [16, 27, 38, 40, 43].

Other factors such as personal values and beliefs of the workforce can also influence the perception of work dignity and decent work. For example, personal commitment to the profession and organization of nursing, psychological capital, work engagement, willingness to adopt evidence-based nursing practice, and intention to improve self-professional capabilities (Theme 2) are important influencers of perception about work dignity and decent work at personal level [21, 24, 29–31, 35–37, 42, 46]. The value of workplace social capital is particularly noticed during crises such as the COVID-19 pandemic. Nurses who had a positive perception about their workplace social capital were able to demonstrate stronger moral courage and professional identity [23, 25].

#### 3.3.3. Influence on Partnerships for the Goals (SDG 17)

This goal emphasizes developing innovative strategies for collaboration and cooperation at every level of work settings. Partnership is one of the core pillars of sustainable development and should be initiated at the base of this pillar which is the workplace. We identified two outcomes of nurses' workplace social capital (Theme 2) that are closely related to SDG 17. Trust is the fulcrum for an effective partnership and the tenet of nurses' workplace social capital. Chang et al. [45] documented that nurses who have stronger trust in their workplace are more likely to share their knowledge and tangible and intangible resources with each other. Nurses' workplace social capital also influences effectiveness in dissemination of information and cooperative learning. Pham et al. [32] reported that willingness to mentor and to be mentored increases with constructive workplace social capital network. Nurses' workplace social capital can be perceived as a factor influencing partnerships for the goals (SDG 17).

## 4. Discussion

Nurses by virtue of their professional responsibilities have positive influence in achieving the SDGs of the UN. The precursors to the SDGs can be traced back to Florence Nightingale's remarkable contributions to global health more than a century ago [53]. Nevertheless, nurses may either underestimate and/or misconstrue the strength of their professional power in contributing to the SDGs [7, 49]. Professionally tailored discussions and education are essential in awakening and empowering nurses to assume responsibilities either at the individual level or collectively in the global movement for sustainable development [49, 54, 55].

We conducted this integrative review based on empirical research evidence to address the association between nurses' workplace social capital and the SDGs. We identified 23 outcomes of nurses' workplace social capital which we classified under three themes and have demonstrated their connections and contributions to the goals 3, 8, and 17 of sustainable development (Figure 2 and Table 2).

The core objective of the nursing profession is to promote health for all people, including nurses themselves, at every age and in any setting [6]. Workplace social capital promotes constructive and respectful relational networks between nurses and the other healthcare professionals [11, 15, 56]. These positive interactions reduce the risks of errors in the delivery of healthcare services, improve the practice of shared decision-making in diagnostic and treatment process, and improve the overall quality of care. Furthermore, respectful and trustworthy interactions among nurses and with the other healthcare professionals improve efficiency and effectiveness of the delivery of healthcare services and facilitate constructive clinical management. Ultimately, patients and healthcare providers can benefit from a safer healthcare environment, healthy lives, and overall well-being which are the main objectives of SDG 3 [4].

The high rate of nursing turnover and nursing shortage is a global issue that can have severe economic consequences [57]. For example, the annual economic loss of one percentage increase in nursing turnover rate has been estimated at an average of additional $380,600 per annum for a hospital [58]. In a recent publication, Gilbert discussed the importance of nursing workplace social capital in closing the gap of nursing shortage [12]. Nurses' workplace social capital promotes a supportive and trustworthy work environment which promotes dignity and stability of the workforce and therefore contributes to SDG 8 (decent work and economic growth).

Since the time of Florence Nightingale, nurses have been acting as catalysts in diplomatic, political, and combat arenas, although mostly in marginal positions, to strengthen constructive international partnerships and to reduce human suffering [59]. Establishment of partnerships and cooperation at all levels, SDG 17, is based on mutual trust and leadership. Strengthening nurses' confidence in assuming leadership positions at the national and international levels requires involvement in policy decision-making processes [6, 49]. We argue that nurses' workplace social capital can assist with strengthening of nurses' confidence in seeking leadership positions and diplomatic decision-making processes. However, findings from our present study lack adequate data to support our statement. Future research should shed light on the potential positive effects of nurses' workplace social capital on nurses' confidence in assuming leadership spots and international diplomatic relationship positions.

Finally, we believe nurses' workplace social capital contributes to gender equality (SDG 5). Despite our extensive search, we were not able to identify empirical evidence to support the association between nurses' workplace social capital and gender equality. Nurses despite their long history and contributions to patient care and clinical services, in general, are perceived and treated as “second-class citizens” in the healthcare industry [60, 61]. Women make up 70% of the healthcare professionals across the globe. Yet, they occupy only 25% of all leadership positions. Furthermore, they are paid less than their male counterparts holding similar positions [62]. Meanwhile, gender equality extends beyond women's suffrage and equal pay at work. Nursing historically has been a female-dominant profession; however, since the turn of 21^st^ century more men have been opting to enter this field, despite the implicit cultural stigma [63]. We set forth that the attribute of social cohesion of the nurses' workplace social capital can be a conduit for gender equality. Social cohesion is defined as a resource for generating group unity in nourishing a sense of community among people with diverse backgrounds [14]. We voice that the concept of diverse backgrounds does not preclude gender identity. To our knowledge, presently no research has been published to address the association between nurses' workplace social capital and gender diversity in the nursing profession.

### 4.1. Limitations

Some limitations exist in our integrative review. First, we focused on empirical evidence published in peer-reviewed journals that might not be able to fully demonstrate the contribution of nurses' workplace social capital to the SDGs. Theoretical evidence and other potential resources (e.g., book chapters and grey literature) should be considered in future work. Second, most of the quantitative studies in the literature were conducted with a cross-sectional design. Studies with more robust designs, e.g., longitudinal or interventional, would be beneficial to strengthen our findings. Qualitative studies exploring the benefits of nurses' workplace social capital should be encouraged as we were able to identify only two qualitative studies. Moreover, studies directly addressing the relationships between nurses' workplace social capital and sustainability are suggested. Finally, we only searched articles published in the English language. Evidence published in other languages potentially was missed. Despite these limitations, our integrative review is the first to address the contributions of nurses' workplace social capital to the 17 SDGs of the UN in response to calls for contextualizing and promoting sustainable development. Findings of our integrative review innovatively raise a picture of and clarify dedications of the nurses to a sustainable, peaceful, and healthy world.

## 5. Conclusion

We have conducted an integrative review following the methodology by Whittemore and Knafl [17]. A range of outcomes of nurses' workplace social capital were identified from the empirical evidence and three themes were abstracted. Findings of our review shed light on the significant role of nurses' workplace social capital in attaining three of the SDGs, good health and well-being (SDG 3), decent work and economic growth (SDG 8), and partnerships for the goals (SDG 17). Empirical evidence on the other SDGs, e.g., gender equality (SDG 5), should be explored in future studies. Nurses' depth of knowledge and appreciation of their contributions to the SDGs should be encouraged and facilitated. Our work is the first step in connecting and integrating nurses' workplace social capital to the SDGs in academic context and curriculum. This effort helps nurses to understand their roles and responsibilities in sustainable development in their daily work and is beneficial to the development of nurses' workplace social capital.

## 6. Implication for Nursing Management

Nurses' workplace social capital has become an important concept in the academic domain of nursing management due to its favorable outcomes related to healthcare provisions, workforce, and healthcare organizations. We have made efforts to connect these outcomes to the SDGs. The campaign of sustainable development has offered nurses opportunities to raise their voice at the policy tables; nurses are considered the backbone of healthcare services which is connected to every and all the 17 SDGs. Concrete plans and methodologies that tailor these broad goals to nursing daily work and nursing management are paramount of importance in elevating the role and responsibilities of the nursing professionals. Our work promotes and accelerates this crucial process from a management perspective of nurses' workplace social capital. Education is crucial for integrating nurses into active engagement with the SDGs [49, 50]. The relatively high prevalence of disconnection between the role and responsibilities of nurses and the SDGs among nurses calls for constructive educational interventions. Our findings can be used as educational and management resources in clinical and curricula formulation in academic nursing. Importantly, investments and research on nurses' workplace social capital can be greatly strengthened by connecting and revealing its contributions to the UN universal goals of sustainable development.

## Figures and Tables

**Table 1:** Study characteristics.

Reference	Design	Sample size	Main findings (statistically significant outcomes of nurses' workplace social capital)	Instruments for the main findings
Kida et al. (2023) [21] (Japan)	Cross-sectional survey	659	Workplace social capital had direct effects on affective organizational commitment (*β* = 0.302, *p* < 0.001) and normative organizational commitment (*β* = 0.231, *p* < 0.001)	Bonding Workplace Social Capital Scale; Organizational Commitment Scale in the Human Resource Management checklist
Fisher et al. (2022) [22] (UK)	Qualitative description	8	Social capital (networks and networking) was a factor for positive retention intentions	N/A
Pirdelkhosh et al. (2022) [23] (Iran)	Cross-sectional survey	169	Workplace social capital was positively correlated with moral courage (*r* = 0.29 *p* < 0.01) and happiness (*r* = 0.32, *p* < 0.01) during the COVID-19 pandemic; social capital was a positive predictor of moral courage and happiness (*p* < 0.001)	Onyx and Bullen Social Capital Questionnaire; Moral Courage Questionnaire; Oxford Happiness Inventory
Terri Hinkley (2022) [24] (USA)	Cross-sectional survey	999	Social capital had a direct relationship with second victim severity (*β* = −0.801, *p* < 0.001) and psychological capital (*β* = 0.778, *p* < 0.001)	Social Capital of Nursing (SCON); Second Victim Experience and Support Tool (SVEST); Psychological Capital Questionnaire (PCQ)
Zhang et al. (2021) [25] (mainland China)	Cross-sectional survey	308	Workplace social capital positively predicted professional identity during the COVID-19 outbreak (*β* = 1.26, *p* < 0.001)	Chinese Workplace Social Capital Scale (eight-item measure); Chinese Nurse's Professional Identity Scale
Gensimore et al. (2020) [26] (USA)	Cross-sectional survey	507	Increased social capital led to increased perception of quality of care (*β* = 0.324, *p* < 0.05) and personal accomplishment (*β* = 0.269, *p* < 0.05), and decreased emotional exhaustion (*β* = −0.303, *p* < 0.05) and depersonalization (*β* = −0.186, *p* < 0.05)	A modified Social Capital Scale; the item measuring perception of unit quality (quality of care); Maslach Burnout Inventory
Gholami Motlagh et al. (2020) [27] (Iran)	Cross-sectional survey	99	As the social capital score increased, the job satisfaction score increased (*r* = 0.313, *p*=0.002)	Nahapiet and Ghoshal Social Capital Questionnaire; Minnesota Satisfaction Questionnaire (MSQ)
Hafeez et al. (2020) [28] (Pakistan)	Qualitative description	20	Social capital in the form of social support is a major preventative strategy for workplace occupational injuries and accidents	N/A
Norikoshi et al. (2020) [29] (Japan)	Mixed methods (instrument development and test)	Interviewed 32 nurses for item generation; 414 in the quantitative test stage	Workplace social capital was positively related to work engagement (*r* = 0.36, *p*=0.01) and negatively associated with turnover intention (*r* = −0.40, *p*=0.01)	Relational Workplace Social Capital Scale for Japanese Nurses (RWSCS-JN); Japanese Short Version of the Utrecht Work Engagement Scale; Turnover Intention Scale
Chang et al. (2019) [30] (Taiwan)	Cross-sectional survey	524	Social capital was positively related to normative professional commitment (*β* = 0.34, *p*=0.001)	Modified Contextual Barriers and Supports Measure (Social Capital Subscale); items from Occupational Commitment Scale
Chang et al. (2019) [31] (Taiwan)	Cross-sectional survey	502	Social capital was positively related to intention to improve professional capabilities (path coefficient = 0.21, *p* < 0.05)	Modified Contextual Barriers and Supports Measure (Social Capital Subscale; Choice Goal Subscale)
Pham et al. (2019) [32] (Taiwan)	Cross-sectional survey	166	Mentor-mentee rapport (social capital relationships) was positively related to mentors' willingness to mentor (*β* = 0.85, *p* < 0.001) and mentees' willingness to be mentored (*β* = 0.75, *p* < 0.001)	Adapted items measuring rapport and willingness to mentor/be mentored
Jafari et al. (2018) [33] (Iran)	Cross-sectional survey	215	Social capital was positively related to clinical risk management (*r* = 0.142, *p*=0.040)	Self-developed Social Capital Questionnaire; Risk Management Questionnaire
Middleton et al. (2018) [34] (Cyprus)	Cross-sectional survey	362	Increased odds of mental distress (*OR* = 2.16; 95% CI = 1.05, 4.42) and decreased self-rated health (mean difference = 8.4; 95% CI = 2.8, 14.0) were observed among nurses with the lowest level of workplace social capital	Eight-item Measure of Workplace Social Capital; General Health Questionnaire (GHQ-12); 0–100 Visual Analogue Scale (VAS) on self-rated health
Vagharseyyedin et al. (2018) [35] (Iran)	Cross-sectional survey	250	Workplace social capital could predict affective organizational commitment (*B* = 0.2, *p* < 0.05)	Eight-item Measure of Workplace Social Capital; Allen and Meyer's Affective Commitment Scale (ACS)
Shin and Lee (2017) [36] (South Korea)	Cross-sectional survey	432	Social capital was a predictor of evidence-based practice (EBP) adoption (*F* = 4.393–55.003; *p*=0.001 or <0.001 for different dimensions)	Social Capital of Nursing (SCON); Developing Evidence-Based Practice Questionnaire (DEBPQ)
Van Bogaert et al. (2017) [37] (Belgium)	Mixed methods (explanatory sequential)	751 in the quantitative survey part; 19 in the qualitative stage	Social capital inversely impacted feelings of emotional exhaustion (path coefficient = −0.18, *p* < 0.05) and positively impacted feelings of vigor (path coefficient = 0.23, *p* < 0.05); associations were supported by qualitative findings	Social Capital in Organizations; Maslach Burnout Inventory; Utrecht Work Engagement Scale
Shin and Lee (2016) [38] (South Korea)	Cross-sectional survey	432	Social capital was a positive predictor of job satisfaction (*R*^2^ = 0.501, *p* < 0.001) and quality of care (*R*^2^ = 0.244, *p* < 0.001)	Social Capital of Nursing (SCON); three questions developed by Sheingold and Sheingold on job satisfaction; Service Quality (SERVQUAL)
Farahbod et al. (2015) [39] (Iran)	Cross-sectional survey	214	There was an inverse association between social capital and burnout (*r* = −0.451, *p* < 0.0001); social capital could predict burnout (*β* = −0.34)	Social Capital Questionnaire devised by Boyas and colleagues; Maslach Burnout Inventory
Read and Laschinger (2015) [40] (Canada)	Longitudinal survey	191	Relational social capital had a negative direct effect on mental health symptoms (*β* = −0.21, *p* < 0.05) and a positive direct effect on job satisfaction (*β* = 0.50, *p* < 0.05)	Community Subscale of Areas of Worklife Scale (AWS); Mental Health Inventory (MHI-5); items from Shaver and Lacey's scale on job satisfaction
Laschinger et al. (2014) [41] (Canada)	Cross-sectional survey	525	Social capital had direct effects on unit effectiveness (*β* = 0.29, *p* < 0.05) and quality of care (*β* = 0.21, *p* < 0.05)	Items from Shortell Organizational Culture Scale (social capital and unit effectiveness); a single item developed in the Magnet hospital studies (quality of care)
Van Bogaert et al. (2014) [16] (Belgium)	Cross-sectional survey	1,108	Social capital was related to job satisfaction (OR = 2.19; 95% CI = 1.39, 3.45), (no) intention to leave (OR = 2.07, 95% CI = 1.36, 3.16), quality of care (OR = 13.18, 95% CI = 7.85, 22.92), and medication errors (OR = 0.61, 95% CI = 0.43, 0.86)	Social Capital in Organizations; self-developed measures on job outcome (job satisfaction and intention to leave), quality of care, and adverse patient events (medication errors)
Van Bogaert et al. (2014) [42] (Belgium)	Cross-sectional survey	1,201	Social capital had a statistically significant effect on vigor in the improved structural equation model (path coefficient = 0.24)	Social Capital in Organizations; Utrecht Work Engagement Scale
Sheingold and Sheingold (2013) [43] (USA)	Mixed methods (instrument development and test)	Focus groups with 80 nurses for item generation; 325 in the quantitative test stage	Social capital had positive impacts on job satisfaction (*R*^2^ = 0.557, *p*=0.000) and intention to stay (*R*^2^ = 0.345, *p*=0.000)	Social Capital of Nursing (SCON); self-developed measures on job satisfaction and intention to stay
Van Bogaert et al. (2013) [44] (Belgium)	Cross-sectional survey	1,201	A direct path was accepted between social capital and emotional exhaustion in the improved structural equation model (path coefficient = −0.16, *p* < 0.05)	Social Capital in Organizations; Maslach Burnout Inventory
Chang et al. (2012) [45] (Taiwan)	Cross-sectional survey	797	Trust (*β* = 0.220, *p* < 0.01) and shared vision (*β* = 0.184, *p* < 0.05) of social capital directly affected knowledge sharing; shared vision had a significant effect on patient safety (*β* = 0.195, *p* < 0.05)	Items from three valid scales (social capital); items adapted from van den Hooff and van Weenen on knowledge sharing; indicators proposed by JCAHO on patient safety
Hsu et al. (2011) [46] (Taiwan)	Cross-sectional survey	797	Social capital (social interaction (*β* *=* 0.13, *p* < 0.05), trust (*β* *=* 0.27, *p* < 0.01), and shared vision (*β* *=* 0.34, *p* < 0.001)) positively impacted organizational commitment	Measures adapted from several valid instruments
Kowalski et al. (2010) [47] (Germany)	Cross-sectional survey	959	Social capital was negatively associated with emotional exhaustion (OR = 0.549, 95% CI = 0.403, 0.746)	Social Capital in Organizations; Maslach Burnout Inventory
Ernstmann et al. (2009) [48] (Germany)	Cross-sectional survey	959	Higher social capital was associated with better clinical risk management behavior in the hospital (intercorrelation coefficient = 0.472, *p*=0.01)	Social Capital in Organizations; Risk Management Questionnaire

**Table 2:** Influence of nurses' workplace social capital on the sustainable development goals (SDGs).

Outcomes of nurses' workplace social capital	Number of outcomes	Themes	Main influence on the SDGs
Quality of care [16, 26, 38, 41] Patient safety [16, 45]	2	Theme 1	SDG 3: good health and well-being
Happiness [23] Self-rated health [34] Mental distress^∗^ [34, 40] Second victim severity^∗^ [24] Burnout^∗^ [26, 37, 39, 44, 47]	5	Theme 2	
Preventative strategy for occupational injuries and accidents [28] Clinical risk management [33, 48] Unit effectiveness [41]	3	Theme 3	
Organizational commitment [21, 35, 46] Professional commitment [30] Retention intentions/intention to stay [16, 22, 43] Turnover intention^*∗*^ [29] Moral courage [23] Psychological capital [24] Professional identity [25] Job satisfaction [16, 27, 38, 40, 43] Work engagement [29, 37, 42] Intention to improve professional capabilities [31] Evidence-based practice adoption [36]	11	Theme 2	SDG 8: decent work and economic growth
Willingness to mentor/be mentored [32] Knowledge sharing [45]	2	Theme 2	SDG17: partnerships for the goals
Total	23		

Note: Theme 1: healthcare services management; Theme 2: workforce management; Theme 3: workplace management. ^*∗*^Nurses' workplace social capital attenuates the level of mental distress, second victim severity, burnout, and turnover intention.

## Data Availability

Supporting data for this study are included within the article and its supplementary tables.

## Supplementary Materials

Supplementary Materials Supplementary Table 1: Search strategies. Supplementary Table 2: Quality assessment of the included articles.
