# Supplementary material for: Nurses' Workplace Social Capital and Sustainable Development: An Integrative Review of Empirical Studies
Source: J Nurs Manag. 2024 Jul 17;2024:8362035. doi: 10.1155/2024/8362035 (PMC11918928; doi:10.1155/2024/8362035)
Supplement: Supplementary Materials — Supplementary Table 1: Search strategies. Supplementary Table 2: Quality assessment of the included articles. [file 8362035.f1.docx]

| Supplementary Table 1: Search strategies | | |
| --- | --- | --- |
| 1) **Medline**: 242 hits | | |
| ("Nurses"[MeSH] OR "Nursing"[MeSH] OR "Nurses"[Title/Abstract] OR "Nursing"[Title/Abstract] OR "Nurse"[Title/Abstract]) AND ("Social Capital"[Mesh] OR "Social Capital"[Title/Abstract]) | | |
| 2) **CINAHL Plus**: 843 hits | | |
| # | Searches | Results |
| S1 | (MH "Nurses+") | 228,667 |
| S2 | TX ( "Nurses" OR "Nursing" OR "Nurse" ) | 1,962,250 |
| S3 | S1 OR S2 | 1,962,323 |
| S4 | (MH "Social Capital") | 2,970 |
| S5 | TX ("Social Capital" ) | 4,553 |
| S6 | S4 OR S5 | 4,553 |
| S7 | S3 AND S6 | 843 |
| 3) **Web of Science**: 282 hits | | |
| TS=( ("Nurses" OR "Nursing" OR "Nurse") AND ("Social Capital") ) | | |
| 4) **Cochrane**: 68 hits | | |
| # | Searches | Results |
| 1 | MeSH descriptor: [Nursing] explode all trees | 4,621 |
| 2 | MeSH descriptor: [Nurses] explode all trees | 1,602 |
| 3 | 'Nurses' OR 'Nursing' OR 'Nurse' | 71,944 |
| 4 | 1 OR 2 OR 3 | 72,215 |
| 5 | MeSH descriptor: [Social Capital] explode all trees | 34 |
| 6 | "Social Capital" | 252 |
| 7 | 5 OR 6 | 252 |
| 8 | 4 AND 7 | 68 |
| 5) **Embase**: 262 hits | | |
| **#** | Searches | Results |
| 1 | 'nurse'/exp | 220,104 |
| 2 | 'nursing'/exp | 423,128 |
| 3 | 'nurses':ti,ab,kw OR 'nursing':ti,ab,kw OR 'nurse':ti,ab,kw | 628,257 |
| 4 | 1 OR 2 OR 3 | 859,211 |
| 5 | 'social capital'/exp | 3,998 |
| 6 | 'social capital':ti,ab,kw | 5,529 |
| 7 | 5 OR 6 | 6,154 |
| 8 | 4 AND 7 | 262 |
| 1. **PsycINFO**: 132 hits | | |
| # | Searches | Results |
| 1 | DE "Nurses" | 34,258 |
| 2 | DE "Nursing" | 27,643 |
| 3 | TI ( 'Nurses' OR 'Nursing' OR 'Nurse' ) OR AB ( 'Nurses' OR 'Nursing' OR 'Nurse' ) OR KW ( 'Nurses' OR 'Nursing' OR 'Nurse' ) | 114,763 |
| 4 | 1 OR 2 OR 3 | 115,615 |
| 5 | DE "Social Capital" | 7,662 |
| 6 | TI "Social Capital" OR AB "Social Capital" OR KW "Social Capital" | 9,897 |
| 7 | 5 OR 6 | 10,732 |
| 8 | 4 AND 7 | 132 |
| 7) **Scopus**: 359 hits | | |
| TITLE-ABS-KEY(("Nurses" OR "Nursing" OR "Nurse") AND TITLE-ABS-KEY("Social Capital")) | | |

*Note:* Final searching date: May 31^th^, 2023.

Supplementary Table 2: Quality assessment of the included 29 articles using the Mixed Methods Appraisal Tool (MATT, Hong et al., 2018)

| **Reference** | **Methodological quality criteria** | | | | |
| --- | --- | --- | --- | --- | --- |
|  | **Question**  **1** | **Question**  **2** | **Question**  **3** | **Question**  **4** | **Question**  **5** |
| **Qualitative** | Is the qualitative approach appropriate to answer the research question? | Are the qualitative data collection methods adequate  to address the research question? | Are the findings adequately derived from the data? | Is the interpretation of results sufficiently substantiated by data? | Is there coherence between qualitative data sources, collection, analysis and interpretation? |
| Fisher et al., 2022 [22] | Yes | Yes | Yes | Yes | Yes |
| Hafeez et al. 2020 [28] | Yes | Yes | Yes | Yes | Yes |
| **Quantitative** | Is the sampling strategy relevant to address the research question? | Is the sample representative of the target population? | Are the measurements appropriate? | Is the risk of nonresponse bias low? | Is the statistical analysis appropriate to answer the research question? |
| Kida et al., 2023 [21] | Can’t tell | Yes | Yes | Can’t tell | Yes |
| Pirdelkhosh et al., 2022 [23] | Yes | Yes | Yes | Yes | Yes |
| Terri Hinkley, 2022 [24] | Yes | Yes | Yes | Yes | Yes |
| Zhang et al., 2021 [25] | Can’t tell | Yes | Yes | Yes | Yes |
| Gensimore et al., 2020 [26] | Can’t tell | Yes | Yes | Yes | Yes |
| Gholami Motlagh et al., 2020 [27] | Yes | Yes | Yes | Yes | Yes |
| Chang et al, 2019 [30] | Yes | Yes | Yes | Yes | Yes |
| Chang et al, 2019 [31] | Yes | Yes | Yes | Yes | Yes |
| Pham et al., 2019 [32] | Yes | Yes | Yes | Yes | Yes |
| Jafari et al., 2018 [33] | Can’t tell | Can’t tell | Can’t tell | Yes | Yes |
| Middleton et al., 2018 [34] | Yes | Yes | Yes | Yes | Yes |
| Vagharseyyedin et al., 2018 [35] | No | Yes | Yes | Can’t tell | Yes |
| Shine and Lee, 2017 [36] | Yes | Yes | Yes | Yes | Yes |
| Shine and Lee, 2016 [38] | Yes | Yes | Yes | Yes | Yes |
| Farahbod et al., 2015 [39] | Can’t tell | Can’t tell | Can’t tell | Yes | Yes |
| Read and Laschinger, 2015 [40] | Yes | Yes | Yes | Can’t tell | Yes |
| Laschinger et al., 2014 [41] | Yes | Yes | Yes | Can’t tell | Yes |
| Van Bogaert et al., 2014 [16] | Yes | Yes | Yes | Yes | Yes |
| Van Bogaert et al., 2014 [42] | Can’t tell | Yes | Yes | Yes | Yes |
| Van Bogaert et al., 2013 [44] | Yes | Yes | Yes | Yes | Yes |
| Chang et al., 2012 [45] | Yes | Yes | Yes | Yes | Yes |
| Hsu et al., 2011 [46] | Yes | Yes | Yes | Yes | Yes |
| Kowalski et al., 2010 [47] | Yes | Yes | Yes | Yes | Yes |
| Ernstmann et al., 2009 [48] | Yes | Yes | Yes | Yes | Yes |
| **Mixed methods** | Is there an adequate rationale for using a mixed methods design to address the research question? | Are the different components of the study effectively integrated to answer the research question? | Are the outputs of the integration of qualitative and quantitative components adequately interpreted? | Are divergences and inconsistencies between quantitative and qualitative results adequately addressed? | Do the different components of the study adhere to the quality criteria of each tradition of the methods involved? |
| Norikoshi et al., 2020 [29] | Yes | Yes | Yes | Can’t tell | Can’t tell |
| Van Bogaert et al., 2017 [37] | Yes | Yes | Can’t tell | Yes | Yes |
| Sheingold and Sheingold, 2013 [43] | Yes | Yes | Yes | Can’t tell | Can’t tell |

Q. N. Hong, P. Pluye, S. Fàbregues, G. Bartlett, F. Boardman, M. Cargo, P. Dagenais, M.-P. Gagnon, F. Griffiths, B. Nicolau, A. O’ Cathain, M.-C. Rousseau, and I. Vedel, “Mixed methods appraisal tool (MMAT), version 2018,” 2018.
